# The use of machine learning and deep learning techniques to assess proprioceptive impairments of the upper limb after stroke

**DOI:** 10.1186/s12984-023-01140-9

**Published:** 2023-01-27

**Authors:** Delowar Hossain, Stephen H. Scott, Tyler Cluff, Sean P. Dukelow

**Affiliations:** 1 Department of Clinical Neuroscience, Cumming School of Medicine, University of Calgary, Calgary, AB Canada; 2 Faculty of Kinesiology, University of Calgary, Calgary, AB Canada; 3 Department of Biomedical and Molecular Sciences, Queen’s University, Kingston, ON Canada

**Keywords:** Stroke, Proprioception, Robotics, Position sense, Machine learning, Deep learning

## Abstract

**Background:**

Robots can generate rich kinematic datasets that have the potential to provide far more insight into impairments than standard clinical ordinal scales. Determining how to define the presence or absence of impairment in individuals using kinematic data, however, can be challenging. Machine learning techniques offer a potential solution to this problem. In the present manuscript we examine proprioception in stroke survivors using a robotic arm position matching task. Proprioception is impaired in 50–60% of stroke survivors and has been associated with poorer motor recovery and longer lengths of hospital stay. We present a simple cut-off score technique for individual kinematic parameters and an overall task score to determine impairment. We then compare the ability of different machine learning (ML) techniques and the above-mentioned task score to correctly classify individuals with or without stroke based on kinematic data.

**Methods:**

Participants performed an Arm Position Matching (APM) task in an exoskeleton robot. The task produced 12 kinematic parameters that quantify multiple attributes of position sense. We first quantified impairment in individual parameters and an overall task score by determining if participants with stroke fell outside of the 95% cut-off score of control (normative) values. Then, we applied five machine learning algorithms (i.e., Logistic Regression, Decision Tree, Random Forest, Random Forest with Hyperparameters Tuning, and Support Vector Machine), and a deep learning algorithm (i.e., Deep Neural Network) to classify individual participants as to whether or not they had a stroke based only on kinematic parameters using a tenfold cross-validation approach.

**Results:**

We recruited 429 participants with neuroimaging-confirmed stroke (< 35 days post-stroke) and 465 healthy controls. Depending on the APM parameter, we observed that 10.9–48.4% of stroke participants were impaired, while 44% were impaired based on their overall task score. The mean performance metrics of machine learning and deep learning models were: accuracy 82.4%, precision 85.6%, recall 76.5%, and F1 score 80.6%. All machine learning and deep learning models displayed similar classification accuracy; however, the Random Forest model had the highest numerical accuracy (83%). Our models showed higher sensitivity and specificity (AUC = 0.89) in classifying individual participants than the overall task score (AUC = 0.85) based on their performance in the APM task. We also found that variability was the most important feature in classifying performance in the APM task.

**Conclusion:**

Our ML models displayed similar classification performance. ML models were able to integrate more kinematic information and relationships between variables into decision making and displayed better classification performance than the overall task score. ML may help to provide insight into individual kinematic features that have previously been overlooked with respect to clinical importance.

**Supplementary Information:**

The online version contains supplementary material available at 10.1186/s12984-023-01140-9.

## Introduction

Proprioception is the sense of body position, motion, and force based on information from muscle spindles, Golgi tendon organs, cutaneous receptors, joint receptors, and efference copy of motor commands [1–4]. Proprioceptive impairments are common after stroke [5–7] and occur in as many as 64% of stroke survivors [8]. These impairments are associated with deficits in learning sequences of movements [9], as well as decreased independence, quality of life, and poor functional recovery [10].

Clinical assessments of proprioception have traditionally relied on coarse observer-based examinations. Most often, patients are asked to close their eyes while an examiner moves the distal part of the patient’s finger, or the entire finger, up or down. The patient is asked to report the position of their fingertip/finger. Alternatively, some clinicians administer the Thumb Localization Test [11]. This is a simple test in which the clinician passively moves the patient’s hand to a random position overhead while the patient’s eyes are closed, and the patient must then reach to grasp their passively moved thumb with the opposite hand. Unfortunately, these clinical tests have poor reliability, lack resolution, and display ceiling effects [12, 18–20]. Some research groups have designed standardized clinician-administered tests such as the Nottingham Sensory Assessment [12], Wrist Position Sense Test (WPST) [13, 14], and Rivermead Assessment of Somatosensory Performance (RASP) [15] in attempts to deal with the issues outlined above.

However, much of the field studying proprioception has moved to the use of automated measurement tools [16, 17]. Robotic and instrumented assessments are commonly used in research studies of upper extremity proprioception [13, 21–23]. Some authors have used passive movement threshold detection paradigms [24, 25], whereas others have used single limb position-matching [26–29] or mirror-matching tasks [29–31]. Our group has significant experience using a robotic arm position matching task in individuals after stroke [31–34]. The arm position matching task, which we used in this study, relies on mirror matching and can measure various aspects of an individual’s position sense, including variability in matching positions, systematic shifts in the perceived workspace, and perceived contraction or expansion of the workspace. This task can be administered quickly (~ 3 min) and has several advantages over typical clinical measures, including generating reliable, continuous measures of position sense after stroke, lack of floor or ceiling effects, and the fact that the interpretation of human examiners is not required [31]. Robotic proprioceptive testing produces a rich dataset of kinematic measures that quantify impairments that can be difficult to assess at the bedside by a clinician observer.

Robotic assessments of proprioception can generate a large volume of data that may eventually be useful in predicting outcomes and planning for treatment after stroke. Machine Learning (ML) may be helpful in this regard. Several different ML methods exist, each taking advantage of different mathematical processes and algorithms. Some ML methods are more appropriate for certain types of data [35, 36]. Several past studies have attempted to predict clinical outcomes following stroke (e.g., discharge from a rehabilitation unit to home, risk of medical complications, risk of readmission to hospital) using standardized observer-based clinical scales [37–43]. Many of these studies relied on Logistic Regression, although a few used Machine Learning techniques [53–56]. ML techniques are highly effective algorithms that are driven by large volumes of data and can aid in prognosis. They are a set of powerful algorithms capable of modeling hidden and complex relationships between clinical variables and treatment outcomes without necessarily relying on any formal statistical assumptions [44]. Recently, Deep Learning-based approaches such as Deep Neural Networks, a broader family of ML techniques, have achieved impressive results across a variety of Artificial Intelligence (AI) fields [45–49]. Deep Learning approaches are inspired by the human brain’s ability to abstract high-level representations from low-level sensory stimuli [50]. These multi-leveled approaches can be mathematically represented as multi-layered neural networks and recently are able to be trained in layer-wise backpropagation to obtain tractable optimization [51]. These techniques are currently state-of-the-art in applications of speech recognition, image processing, computer vision, and natural language processing [52].

ML techniques have begun to receive some attention in the field of stroke recovery. ML has been used for predictive modelling of recovery using the Barthel Index [57] and to help interpret data collected from Inertial Measurement Units in post-stroke gait [58]. There are significant opportunities for the use of ML techniques as interest continues to grow in the use of kinematic measures to quantify sensory and motor functions. Identifying particular kinematic attributes that may not be easily recognized on clinical examination, predicting recovery following stroke and/or identifying specific kinematic attributes that might be important for targeted intervention would seem to be potential uses of ML in stroke recovery. In the current study, we sought to examine the ability of ML techniques to use kinematic data to determine whether or not an individual had a stroke. Our experimental example was chosen to provide a good test of the ability of different ML techniques to use kinematic data to classify stroke history when the outcome was known. We chose to do this with data from a proprioceptive task, as clinical assessments can be unreliable and making such a determination based on clinical data or individual kinematic parameters could prove to be substantially challenging. Further, post-stroke proprioceptive deficits can significantly impact stroke recovery. Studies have linked impaired proprioception with poorer motor recovery [59–63] and longer lengths of hospital stay [64]. Given the challenging nature of quantifying proprioception, we felt it may provide an ideal test case for the utility of ML techniques. Success at using complex kinematics from a robotic proprioceptive task to classify stroke, could bode well for future more challenging classification tasks in other neurologic conditions where the diagnosis is not yet known.

In the present study, we examined the performance of individuals with stroke and healthy controls on a robotic Arm Position Matching (APM) task. The goals of this study were: (1) to compare the different ML and DL techniques with a more traditional model that relied on the 95% cut-off score of normative data for different attributes of position sense, determining which technique flagged the highest number of stroke participants as abnormal, (2) to compare different ML and DL techniques, and their ability to classify whether someone has had a stroke or not, and (3) to examine the relative importance of different parameters measured in the APM task and their usefulness in classifying whether or not someone has had a stroke.

## Methods

### Participants

Participants with stroke were recruited from the inpatient acute stroke or stroke rehabilitation units at the Foothills Medical Centre, the Dr. Vernon Fanning Care Centre in Calgary, Alberta, Canada, and Providence Care, St Mary’s of the Lake Hospital, Kingston, Ontario, Canada. Inclusion criteria for participants with stroke were: recent onset (< 35 days) of first clinical stroke and age ≥ 18 years. Exclusion criteria for participants with stroke were: other underlying neurological conditions (e.g. Parkinson’s, Multiple Sclerosis), upper limb orthopedic impairments, inability to understand task instructions or evidence of apraxia [65]. Neurologically intact control participants who also met the inclusion and exclusion criteria above, but had no history of stroke, were recruited from the communities of Calgary, Alberta, and Kingston, Ontario, Canada. This study was reviewed and approved by the University of Calgary Conjoint Health Research Ethics Board and the Queen’s University Research Ethics Board. All participants gave written informed consent before performing the assessment.

### Robotic assessment

**Fig. 1:**
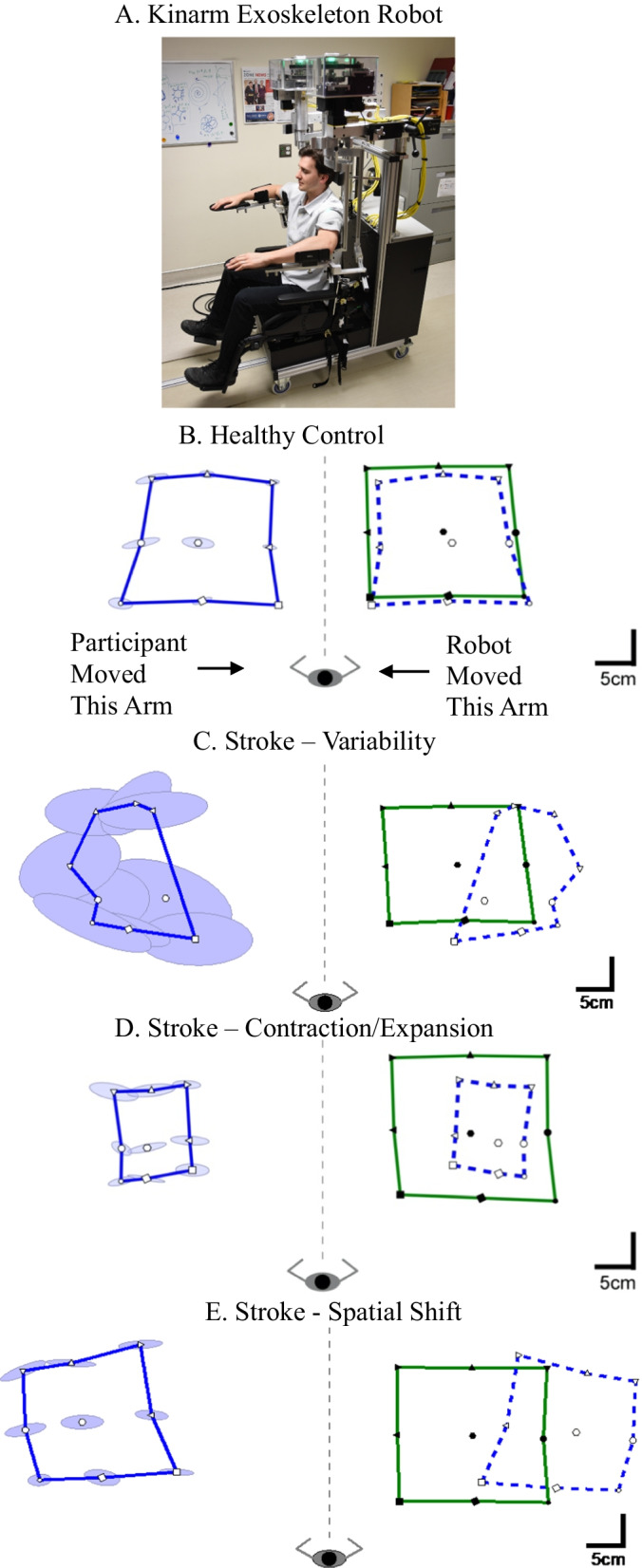
Arm Position Matching (APM) task. **A** The Kinarm exoskeleton robot. **B** Typical healthy control participant data. The robot moved the participant’s passive right hand to one of 9 spatial locations (filled symbols). The participant then attempted to mirror match with the left active hand (open symbols). The solid blue line connects the average final positions of the outer eight target locations of the matching hand (active hand). Solid green line connects the outer eight targets for the robot moved passive hand. The dashed blue line is the mirror reflection of solid blue line, which allows a visual comparison of the average final outer 8 positions of the active and passive (robot-moved) hands. Ellipses represent one standard deviation of the matched positions. The ellipses represent trial-to-trial variability, where a larger ellipse means the participant was less consistent (i.e., more variable) in matching the position of their passive hand with the active hand. **C** An exemplar stroke participant who demonstrated high variability in position matching. **D** An exemplar stroke participant who demonstrated a contracted sense of their workspace. **E** An exemplar stroke participant who demonstrated a spatial shift of their workspace

*Robotic Device* The robotic assessment of position sense was performed using a Kinarm Exoskeleton robotic device [66] (Fig. 1A; Kinarm, Kingston, Ontario, Canada), which permits movements of the arm in the horizontal plane involving horizontal abduction/adduction of the shoulder and flexion/extension of the elbow. The planar robot has 2 degrees of freedom, articulating at both the shoulder and elbow. The device records at a rate of 1000 Hz and has a position resolution of 0.1 mm. Participants were seated in a height-adjustable wheelchair base with their arms supported against gravity. The device was fit to each participant’s arm by research staff who were trained to conduct the robotic assessment. The robot was wheeled to a 2D virtual/augmented reality display. The visual display is capable of projecting virtual targets into the plane of the participant’s arm during calibration and task performance. Given the focus on proprioceptive function, visual stimuli were not displayed on the screen during the experiment. Direct vision of the upper extremities was occluded by a shutter and a bib. The set-up and calibration procedures took between 6 and 8 min for each participant.

*Arm Position Matching Task* The Arm Position Matching (APM) task was used to assess the individual’s position sense of their arm and has been described previously [31–34, 67]. Participants were instructed to relax one arm (passive hand) and let the robot passively move their hand to one of four/nine spatial locations separated by 20/10 cm (Fig. 1B, 9-target task). The 4-target protocol is spaced on a 2 × 2 grid with targets spaced at 20 cm intervals in the X- and Y-directions. The 9-target protocol is the same as the 4-target protocol but includes nine targets spaced on a 3 × 3 grid at 10 cm intervals. Target locations were pseudo-randomized within a block. Each block contained one trial at each target location and participants completed six blocks. The robot moved the passive hand using a bell-shaped speed profile (max speed < 1 m/s). After the robot completed the passive movement, participants were asked to move their other arm (active hand; Fig. 1B) to mirror-match the spatial position of the passive hand. Participants were granted as much time as necessary to match the active hand position with the passive hand. Participants notified the examiner when they had matched their hand position, and the examiner triggered the next trial. Each participant completed either the 4-target or 9-target task protocol [68]. For the stroke participants, the affected arm was always the passive hand. Healthy control participants completed the task twice, where each arm served as the passive hand once and we consider data from each arm as a separate participant in the analysis [69].

### Robotic task parameters

The following parameters were used to quantify task performance after completing all trials: (a) trial-to-trial *Variability* (Var) of the active hand, (b) *Spatial Contraction/Expansion* *Ratio* (Cont/Exp) of the area matched by the active hand, (c) *Systematic Spatial Shifts* (Shift) between the passive and active hands, and (d) *Absolute Error* (AE).

*Variability*: *Variability* in Arm Position Matching (APM) describes the trial-to-trial consistency of the active hand in matching the same target position (Fig. 1C). It was calculated as the standard deviation of the active hand’s position for each target location. The mean of the standard deviations was then calculated across all target positions in the x-coordinate (*Var*_x_), y-coordinate (*Var*_y_), and resultant linear variability of both coordinates (*Var*_xy_):

1 $${Var}_{xy}=\sqrt{{{Var}_{x}}^{2}+{{Var}_{y}}^{2}}$$

*Spatial Contraction/Expansion Ratio*: Spatial *Contraction/Expansion Ratio* describes whether a participant displayed contraction or expansion of their perceived workspace (Fig. 1D). It was calculated as the matched area/range of the workspace of the active hand relative to the passive hand. This parameter was calculated for the matched x-coordinates (*Cont/Exp*_*x*_) by finding the difference between the mean x-coordinate of the three left and three right targets for the active hand compared with the passive hand:

2 $${Cont/Exp}_{x}=\frac{{range}_{{x}_{active}}}{{range}_{{x}_{passive}}}$$

A similar procedure was used to calculate contraction/expansion in the y-coordinate (*Cont/Exp*_*y*_) using the range of the top and bottom three targets. Spatial contraction/expansion in both the x- and y- coordinates (*Cont/Exp*_*xy*_) was calculated by finding the area spanned by the active hand for the eight border targets and then normalized by the total spatial area spanned by these same targets using the passive hand.

*Systematic Spatial Shifts*: Systematic Spatial *Shifts* describe constant errors between the active and passive hands (Fig. 1E). These errors were calculated as the mean error between the passive and active hands for each target position. The mean was then calculated using the means for all target locations. Systematic shifts were calculated in the x-coordinate (*Shift*_*x*_), y-coordinate (*Shift*_*y*_), and combined across both coordinates to provide a measure of the resultant shift in matched positions (*Shift*_*xy*_):

3 $${Shift}_{xy}=\sqrt{{{Shift}_{x}}^{2}+{{Shift}_{y}}^{2}}$$

*Absolute Error*: *Absolute Error* describes the mean absolute distance error between the position of the active and passive hands. The mean absolute distance error between the active hand and the target position was calculated across all trials in the x-coordinate (*AE*_*x*_), y-coordinate (*AE*_*y*_), and combined across both coordinates (*AE*_*xy*_):

4 $${AE}_{xy}=\sqrt{{{AE}_{x}}^{2}+{{AE}_{y}}^{2}}$$

A total of 12 parameters were used to measure performance in the arm position matching task.

*Z-score*: For each of the parameters above, we relied on the Dexterit-E software [70] associated with the Kinarm to calculate a Z-score. The Z-score or standardized score, is the distance, measured in standard deviations, that a data point falls from the mean of the healthy cohort. Kinarm (Kinarm, Kingston, ON) [71] uses a consistent methodology for developing normal models to calculate the Z-scores of each parameter. Parameter scores from the distribution of the normative data set (developed from neurologically intact controls) are transformed using a Box-Cox power transformation to convert the distribution to a normal distribution [72]. The transformed data are fitted by accounting for age, sex, handedness, and robotic platform (exoskeleton, endpoint robot) using Multiple Linear Regression (MLR). After the first regression, the standard deviation of the residuals is then modeled using a second MLR accounting for the same factors (age, sex, handedness, robotic platform). Z-scores are calculated using the residuals of the first regression and standard deviation modeled by second regression for each parameter. Z-scores are the particular values from the mean, i.e., a Z-score of 1 signifies that a value was 1 standard deviation above the mean, and a Z-score of − 1 signifies that a value was 1 standard deviation below the mean of the healthy control data.

To ensure the distribution was “close-to-normal”, the skew and kurtosis of the final distribution were calculated and compared to the following criteria (Eqs. 5 and 6). These criteria were selected from Pearson and Please [73] so that it is statistically valid to use parametric tests with the Z-scores.

5 $$skew: abs(\sqrt{{\beta }_{1}})\le 0.8, \sqrt{{\beta }_{1}}=\frac{{\mu }_{3}}{{\sigma }^{3}}$$

6 $$kurtosis: 2.4\le {\beta }_{2}\le 3.6, {\beta }_{2}=\frac{{\mu }_{4}}{{\sigma }^{4}}$$

where σ is the standard deviation, and ${\mu }_{3}$ and ${\mu }_{4}$ are the third and fourth moments of the mean.

*Task Score*: A task score gives a global measure of a participant’s performance for a given task. It measures how far the participant’s performance is from the best performance. The first stage in calculating the task score is to convert the task parameter scores into standardized Z-scores (described above). The second stage is to identify whether the best performance for a given metric reflects large negative Z-scores, large positive Z-scores, or near zero Z-scores. The Z-scores are transformed into Zeta scores using Eq. 7 for those parameters in which best performance is one-sided (i.e., large negative or large positive Z-scores).

7 $$\varsigma =\surd 2\bullet {erfc}^{-1}(\frac{1}{2}\bullet erfc\left(\frac{\pm z}{\sqrt{2}}\right))$$

where ‘+’ is used when poor performance is positive and ‘-’ is used when poor performance is negative.

In the final stage, task scores are calculated based on the performance of healthy controls. The root-sum-square (RSS) distance of Z-scores and Zeta scores are calculated using Eq. 8 for healthy controls. RSS distance is also known as the Euclidean distance and is transformed into a Z-score using a Box-Cox transform. The Z-score of the RSS distance is then transformed to a one-sided statistic using Eq. 7.

8 $$rssDistance=\sqrt{\sum_{i}{z}_{i}^{2}+\sum_{j}{\varsigma }_{j}^{2}}$$

where $\sum_{i}{z}_{i}^{2}$ includes all two-sided parameters and $\sum_{j}{\varsigma }_{j}^{2}$ includes all one-sided parameters.

Task scores are always positive. A score of 0 corresponds to the best performance, and increasing values represent poorer performance. If the task score is > 3.29 (that is normally 1 in 1000) for control participants, then that participant was classified as an outlier for the task and removed. Outliers were removed to improve the robustness of the modeling process of normative data sets.

### Clinical assessments

A broad range of clinical assessments was performed to characterize the impairment of stroke participants in this study. The assessments served to quantify sensation, movement, cognition, and functional abilities. The assessments were performed by a physician or physiotherapist who had expertise in stroke rehabilitation. They were blinded to the results of the robotic assessment.

Position sense was clinically assessed using the Thumb Localization Test (TLT) [11] because it has been used to quantify whole-limb position sense in many studies involving stroke [74–82]. In this test, the examiner moves the participant’s stroke-affected arm to a position in front of the participant at or above eye level, lateral to the midline with the participant’s eyes closed. The participant is then asked to pinch the thumb of that limb with the opposite thumb and forefinger (reaching limb). Participants were scored as 0 if their performance was considered normal (completed task perfectly) to 3, which is considered markedly abnormal (the participant was unable to find his or her thumb and did not climb up the affected arm to locate it).

Motor impairment was assessed using the Purdue Peg Board test (PPB) (Lafayette Instrument Co., Lafayette, IN, USA) [83] and the Chedoke-McMaster Stroke Assessment (CMSA) [84]. In the PPB assessment, participants placed as many small pegs as possible into holes in a board over 30 s using one hand. The participant is required to use the proximal upper extremity to keep the hand in the appropriate position to retrieve and insert each peg as a test of fine motor skills. The CMSA relies on the concept of stages of motor recovery, which was first introduced by Twitchell [85]. The CMSA classifies participants into subgroups based on the stage of motor recovery. The 7-point scale corresponds to seven stages of motor recovery, where a score of 1 is considered the most abnormal and a score of 7 is normal.

Functional abilities were assessed using the Functional Independence Measure (FIM) [86]. It is used as a metric for independence within activities of daily living. Within the 18-item scale, 13 items are considered motor tasks, and 5 items are considered cognitive tasks. In the current manuscript, we present the total FIM score (measured out of 126) and the FIM score for the motor component (measured out of 91).

### Data analysis

Data analysis was done using Machine Learning and Deep Learning techniques in the Python programming language (version 3.7.4) [87]. In the first step of our analysis, we determined when stroke participants were impaired on robotic parameters using the Z-scores described above. We determined the 95% cut-off score of control performance (Task score > 1.96 is considered as impaired and Task score ≤ 1.96 is considered as unimpaired) on each robotic parameter to find whether an individual participant failed on a given parameter. When a stroke/control participant’s score fell outside of the control range, they were classified as impaired on that robotic task. Our primary analysis compared the impairment rate found on individual parameters and the overall task score (so called cut-off score technique) versus the ability of Machine Learning and Deep Learning techniques to determine impairment. Data imputation was unnecessary as there were no missing data in the sample of control and stroke participants.

### Machine learning and deep learning

**Fig. 2:**
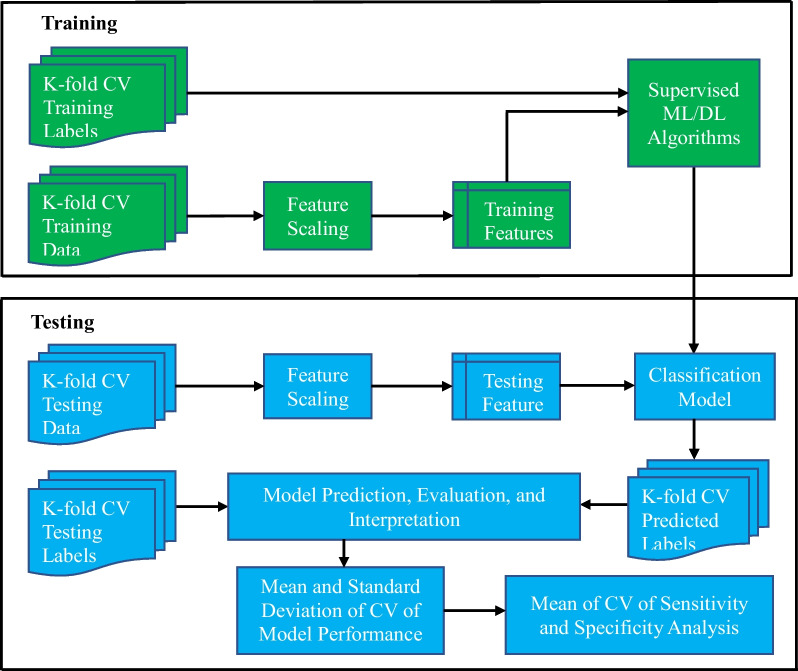
The workflow of K-fold (K = 10) cross-validation (CV) of the machine learning and deep learning models. The training and testing data refer to outcome measures derived from the position matching task in each stroke and control participant. The model generates a label that classified each individual participant as a control or participant with stroke

*Flowchart of the Classification Models*: The workflow blueprint of the data classification models is shown in Fig. 2. The K-fold cross-validation (K = 10, CV) training and testing data represent the outcome measures (features) derived from the Arm Position Matching (APM) task (12 parameters) of each control and stroke participant. K-fold CV training and testing data were classified and labeled into two different categories (“control” and “stroke”). This data was passed through feature extraction and scaling processes. It was then fitted to the supervised machine learning and deep learning models. After evaluation, we calculated the mean and standard deviation across the K-fold CV for all model performance metrics. Finally, we calculated receiver operating characteristic curves (ROC curves) for the mean of the K-fold cross-validated results of each model.

*K-fold Cross-Validation (CV)*: The K-fold Cross-Validation procedure randomly divided the dataset into K-disjoint folds. One-fold was used for testing and remain K-l folds were used for training the model. This process was repeated K-times until the testing was performed on all K folds. All folds contained equal data points unless otherwise specified. We applied K-fold cross-validation (where K = 10) to estimate the performance and reliability of each classification algorithm and enable meaningful comparison between classification models. The performance of the classification models was evaluated by the mean and standard deviation across the K-fold datasets.

*Features*: A feature represents a measurable piece of data that can be used for analysis. It is also known as an “attribute” or “variable”. In our case, features were the Z-score data of the 12 task parameters (Variability X, Variability Y, Variability XY, etc.), such that all features were selected for our analysis. The features were then normalized using the min–max normalization (where the minimum value of that feature got transformed into 0, the maximum value got transformed into 1, and every other value got transformed between 0 and 1) so that the variance of the features was in the same range. Then, features were trained and tested using machine learning and deep learning models. After that, we classified participants with each model and evaluated model performance using the following metrics: accuracy, precision, recall, F1 score, receiver operating characteristics (ROC) curve, and feature importance.

### Classification methods

We applied five Machine Learning (ML) techniques: Logistic Regression (LR) [88], Decision Tree (DT) [89], Random Forest (RF) [90], Random Forest with Hyperparameters Tuning (RFT) [91], and Support Vector Machine (SVM) [92]. We also applied one Deep Learning technique: Deep Neural Network (DNN) [93] for the classification (or supervised learning) of stroke and control data. We chose these 6 techniques as the type of data we were using is compatible with their application. All 6 techniques are commonly used for different ML problems [94–97]. Further, we expected that because of the differences between the techniques, that each technique had the potential to produce a different result when analyzing the same data. We hypothesized that the Random Forest model would outperform the other models because it represents an ensemble learning method rather than a single decision tree [98, 99].

*Logistic Regression (LR)*: We used a Logistic Regression model to classify each participant as a stroke or control based on their performance in the arm position matching task. For that purpose, we implemented a logistic regression classifier that was fitted in the binary logistic regression regularization. This regularization added a penalty as model complexity increased to ensure the model generalized the data and prevented overfitting with an increase in parameters. LR model assumes a linear relationship between the input features and output. The binary logistic model had a dependent variable with two possible outcomes as healthy control and stroke. We used a tolerance of 0.0001 and the maximum number of iterations of 100 as criteria to stop network training.

*Decision Tree (DT)*: We implemented a Decision Tree classifier as one of predictive modeling. It uses a tree-like model in which each internal node (non-leaf) is labeled with an input feature. The arcs coming from a node (branch) labeled with an input feature are labeled with each of the possible values of the target feature or the arcs lead to a subordinate decision node on a different input feature. Each leaf node is labeled with a class either healthy control or stroke. This model splits the nodes of all available features/parameters and then selects the splits, which results in the most homogeneous sub-nodes.

Our decision tree classifier implementation consisted of the following parameters: Gini impurity as a criterion to measure the quality of split, best as a splitter to choose the best split, the maximum depth of the tree as 4, and the minimum number of samples at the leaf node as 1.

*Random Forest (RF)*: We implemented an ensemble learning model (i.e., a Random Forest classifier). It is a classification algorithm consisting of many decision trees, which uses bagging and feature randomness when building each individual tree. It tries to create an uncorrelated forest of trees whose prediction by committee is more accurate than that of any individual tree. The output of the random forest model was the class selected by most trees.

The parameters included in our implementation were: the number of estimators (the number of trees in the forest) was 100, Gini impurity as the criterion for the information gain, the minimum number of samples required to split an internal node was 2, and the minimum number of samples required to be a leaf node was 1. We examined the different number of maximum depths of the tree for the DT model and found that the maximum depth of four gave us the best classification accuracy. The lower number made our model faster, but not as accurate, and higher number gave more accuracy but slow and risk of overfitting.

*Random Forest with Hyperparameters Tuning (RFT)*: We tuned the hyperparameters (a hyperparameter is a parameter whose value is used to control the learning process) of the Random Forest model to determine the best hyperparameters. It relies more on experimental results than theory, and thus the best model to determine the optimal settings was by trying many different combinations to evaluate each model’s performance.

The tuned hyperparameters of the random forest model were: the number of trees in the forest, the maximum number of levels in each decision tree, the maximum number of features considered for spotting a node, the minimum number of data points placed in a node before the node is split, and the minimum number of data points allowed in a leaf node.

*Support Vector Machine (SVM)*: We implemented a Support Vector Machine (SVM) classifier. It constructed a set of hyperplanes (hyperplanes are decision boundaries that help to classify the data points) in high-dimensional space to perform the classification task. The model transformed the data to find an optimal boundary between outputs (control or stroke). A good separation is achieved by the hyperplane that had the largest distance, or functional margin, to the nearest training data point of any class.

Our implementation included the following parameters: the regularization parameter that must be strictly positive, the Radial Basis Function (RBF) type kernel, the size of the kernel cache as 200 MB, the pseudo-random number generator was used for shuffling the data for probability estimators, and tolerance of 0.001 was applied as the network stopping criterion.

*Deep Neural Network (DNN)*: We also implemented a Deep Learning technique, namely, Deep Neural Network (DNN). It is a part of a broader family of machine learning techniques based on artificial neural networks.

Our DNN classifier implementation consisted of three hidden layers between input and output layers. The first hidden layer had 12 units with the Rectified Linear Unit (ReLU) as the activation function, the second hidden layer had 8 units with the ReLU as the activation function, and the third hidden layer had 1 unit with the sigmoid function as the activation function. We also used: binary cross-entropy as loss function, the Root Means Square propagation optimizer (RMSprop), the batch size of 10, and the number epoch of 100. We examined different numbers of epochs (i.e., 50, 100, 200, 500, 1000, 2000, 3000, 5000, 10,000, 20,000, 50,000) for the DNN model and found that 100 epochs gave us the best performance metrics. An epoch refers to the number of passes of the entire training dataset the deep learning technique has completed. The input layer had 12 units for 12 features, and the output had 1 unit to predict a 0 or 1 that maps back to the “healthy control” or “stroke” class. Each layer of nodes trained a distinct set of features based on the output of the previous layer. The feature hierarchical process of our DNN model made it capable of handling very large, and high-dimensional datasets with billions of parameters passed through nonlinear functions.

### Feature importance

Feature importance [100] in machine learning refers to techniques that assign a score to each feature based on their usefulness in the classification task. The score is expressed as a percentage. We applied different feature importance techniques/calculations for the different machine learning techniques. For LR and SVM models, feature importance was based on an information-theoretic criterion, measuring the entropy in the changes of predictions, and perturbation of a given feature [101]. A perturbation, in this instance, is an analytical method to determine an approximate solution of nonlinear equations for which exact solutions cannot be obtained. For the DT, RF, and RFT models, feature importance was computed as the mean and standard deviation of the impurity decrease within each tree [102]. Impurity decrease is the total decrease in node impurity averaged over all ensemble trees and node impurity is a measure of the homogeneity of the labels at a node. In general, a higher score of feature importance means the specific feature has a large effect (importance) on the model that is being used to classify participants as “stroke” and “control”, and a lower score means the specific feature has less impact on the classification model.

## Results

*Participant Demographics*: Data were collected from 429 stroke participants and 465 healthy control participants. Demographics and clinical features of all groups are summarized in Table 1. Ninety-three percent of control and 92% of stroke participants were right-hand dominant. Two control and three stroke participants were scored as having mixed handedness on the Edinburgh Handedness Inventory [103]. Forty-eight percent of stroke participants were observed to have proprioceptive impairment based on the Thumb Localization Test (TLT) test. Seventy-six percent of stroke participants demonstrated motor impairments on their affected arm based on the Chedoke-McMaster Stroke Assessment (CMSA) test.

**Table 1:** Demographic and clinical information for the sample of 894 participants of healthy control and stroke

	Control (n = 465)	Stroke (n = 429)
Age	51 (20–88)	63 (18–92)
Sex	244 M, 221 F	280 M, 149 F
Dominant Hand	434 R, 29 L, 2 A	393 R, 33 L, 3 A
Days since Stroke	$\cdots$	17 (1–34)
Types of Stroke	$\cdots$	370 I, 59 H
TLT [0, 1, 2, 3]		
Affected Side	$\cdots$	[210, 104, 73, 30]*
CMSA [1–7]		
Affected Arm	$\cdots$	[29, 33, 51, 58, 61, 80, 103]
Unaffected Arm	$\cdots$	[0, 0, 0, 0, 15, 94, 320]
CMSA [1–7]		
Affected Hand	$\cdots$	[31, 33, 37, 43, 74, 93, 104]
Unaffected Hand	$\cdots$	[0, 0, 0, 0, 7, 125, 297]
PPB		
Affected Side	$\cdots$	6.9 (0–17.5)
Unaffected Side	$\cdots$	10.5 (2.5–19)
FIM (Total Score)	$\cdots$	93.7 (37–126)
FIM (Motor)	$\cdots$	65 (13–91)

Data are presented as the mean (range) unless otherwise noted. Square brackets for TLT, CMSA scores indicate the actual number of individuals who obtained a given score on the test, e.g., 210 individuals scored 0 on the TLT

M—Male; F—Female; R—Right; L—Light; A—Ambidextrous; H—Hemorrhagic; I—Ischemic; TLT—Thumb Localizing Test; CSMA—Chedoke-McMaster Stroke Assessment; PPB—Purdue Peg Board; and FIM—Functional Independence Measure

*2 scores were missing

**Fig. 3:**
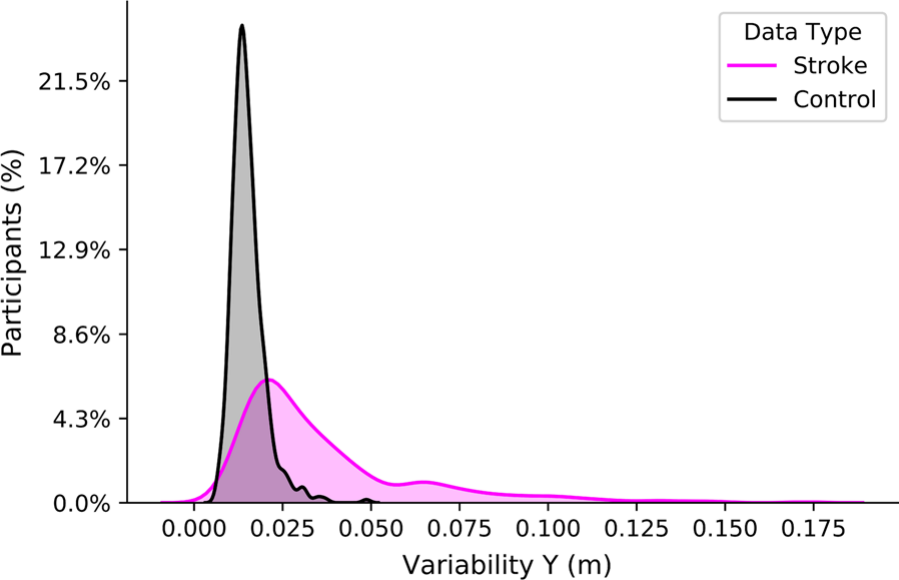
Histogram showing the distribution of *Variability Y* values in healthy controls and participants with stroke. The percentage on the y-axis is the participant count in each bin normalized to the number of participants with stroke (n = 429) and control (n = 465). An Additional file 1: Supplementary figure 1 is available and demonstrates the histogram distribution of all parameters of healthy controls and stroke participants

*Data Visualization*: To visualize the distribution of scores on the robotic task parameters, we plotted histograms of each parameter. *Variability Y* for stroke and control participants is presented in Fig. 3. This exemplar figure demonstrated that the distribution of values of the *Variability Y* parameter overlapped between stroke and control participants. We chose to present *Variability Y* because this parameter had the most influence on the classification tasks (see Fig. 6). Similar findings were seen when examining the distributions of the other parameters (see Additional file 1: Figure S1). The overlap of stroke and control data highlighted the challenge of differentiating normal from abnormal behavior based on a single parameter.

**Fig. 4:**
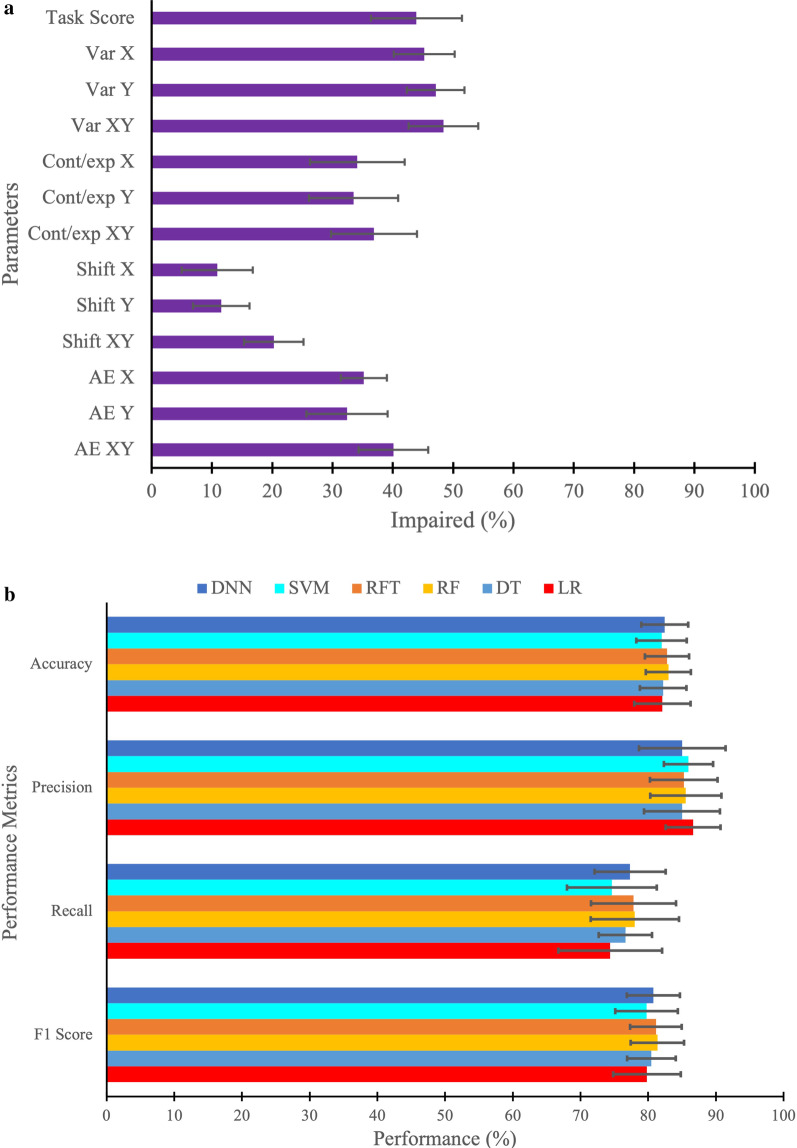
**A** Performance metrics when classified based on individual parameters (Var: variability, Cont/Exp: contraction/expansion, Shift, AE: absolute error) of arm position matching task, as well as overall task score to find the number of impaired participants. **B** Performance metrics (accuracy, precision, recall, and F1 score) for the machine learning and deep learning models (LR: Logistic Regression, DT: Decision Tree, RF: Random Forest, RFT: Random Forest with Hyperparameters Tuning, SVM: Support Vector Machine, DNN: Deep Neural Network)

*Cut-off Score Technique, Machine Learning, and Deep Learning Classifier Models*: We first examined the impairment rates for individual robotic parameters and the overall task score using the 95% cut-off score technique. Figure 4A shows the mean and standard deviation of impairment rates from the tenfold cross-validation of individual parameters (Variability, Contraction/Expansion ratio, Shift, and Absolute Error) and the overall task score to find the number of impaired participants. The result indicates that the highest number of participants were impaired on the parameter *Variability XY* (48.4%) followed by the other two variabilities: *Variability Y* (47.1%) and *Variability X* (45.2%) parameters using the cut-off technique based on individual parameter. The least number of participants were impaired on parameter *Shift X* (10.9%), followed by *Shift Y* (11.5%) and *Shift XY* (20.3%). The overall task score impairment rate was 44% based on the 95% cut-off score technique.

**Fig. 5:**
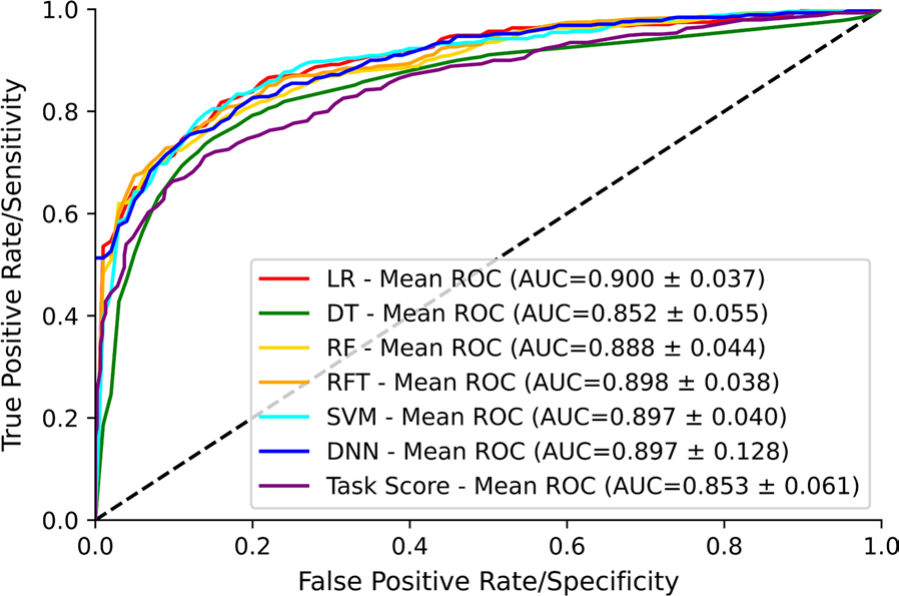
Mean of tenfold cross-validation of the receiver operating characteristic (ROC) curve and area under the curve (AUC) for the classification performance of LR, DT, RF, RFT, SVM, DNN, and task score. The dashed line corresponds to classification due to random chance (AUC = 0.5, i.e., 50% sensitivity and 50% specificity)

We then went on to examine the mean and standard deviation of tenfold cross-validated performance metrics i.e., accuracy, precision, recall, and F1 score [104] of the Machine Learning and Deep Learning models (Fig. 5B). The results of this analysis indicated that RF and RFT had the highest and nearly similar accuracy (83.0% and 82.8%). In the case of precision, LR had a higher value of 86.6% than any other classifier. Again, for recall (78.0%) and F1 score (81.4%) metrics, RF had a higher value of than any other classifier. In terms of standard deviation, LR had the highest spread out over a range of 4.1% for accuracy, 7.8% for recall, and 4.9% for F1 score, whereas DT had the highest spread out over a range of 5.6% for the precision compared with other classifiers.

*Receiver operating characteristic (ROC) curve*: We implemented five Machine Learning classifier models, namely Logistic Regression (LR), Decision Tree (DT), Random Forest (RT), Random Forest with Hyperparameters Tuning (RFT), Support Vector Machine (SVM), and one Deep Learning classifier model, namely Deep Neural Network (DNN) to classify data into two categories: “control” and “stroke”. The mean ROC curve and Area Under the Curve (AUC) value for the classification performance of LR, DT, RF, RFT, SVM, and DNN models is shown in Fig. 5A. We also present the ROC and AUC for the overall task score which is calculated from individual parameter scores as outlined in the methods for comparison. The best possible classifier model would yield a point in the upper-left coordinate (0,1) of the ROC space, representing 100% sensitivity and 100% specificity, which is called a perfect classification. The LR model had the highest AUC (AUC = 0.900) value for the classification task, suggesting that LR had the best separation capability between control and stroke, followed by the RFT model (AUC = 0.898). RFT model performed slightly better at classification than RF (AUC = 0.888). SVM and DNN models performed similarly to the RFT model (AUC = 0.897). The DT performed the worst at the classification task among all models (AUC = 0.852). In summary, LR had the highest level of sensitivity and specificity, whereas DT had the lowest level of specificity and sensitivity among models. For comparison, the overall task score had an AUC = 0.853.

**Fig. 6:**
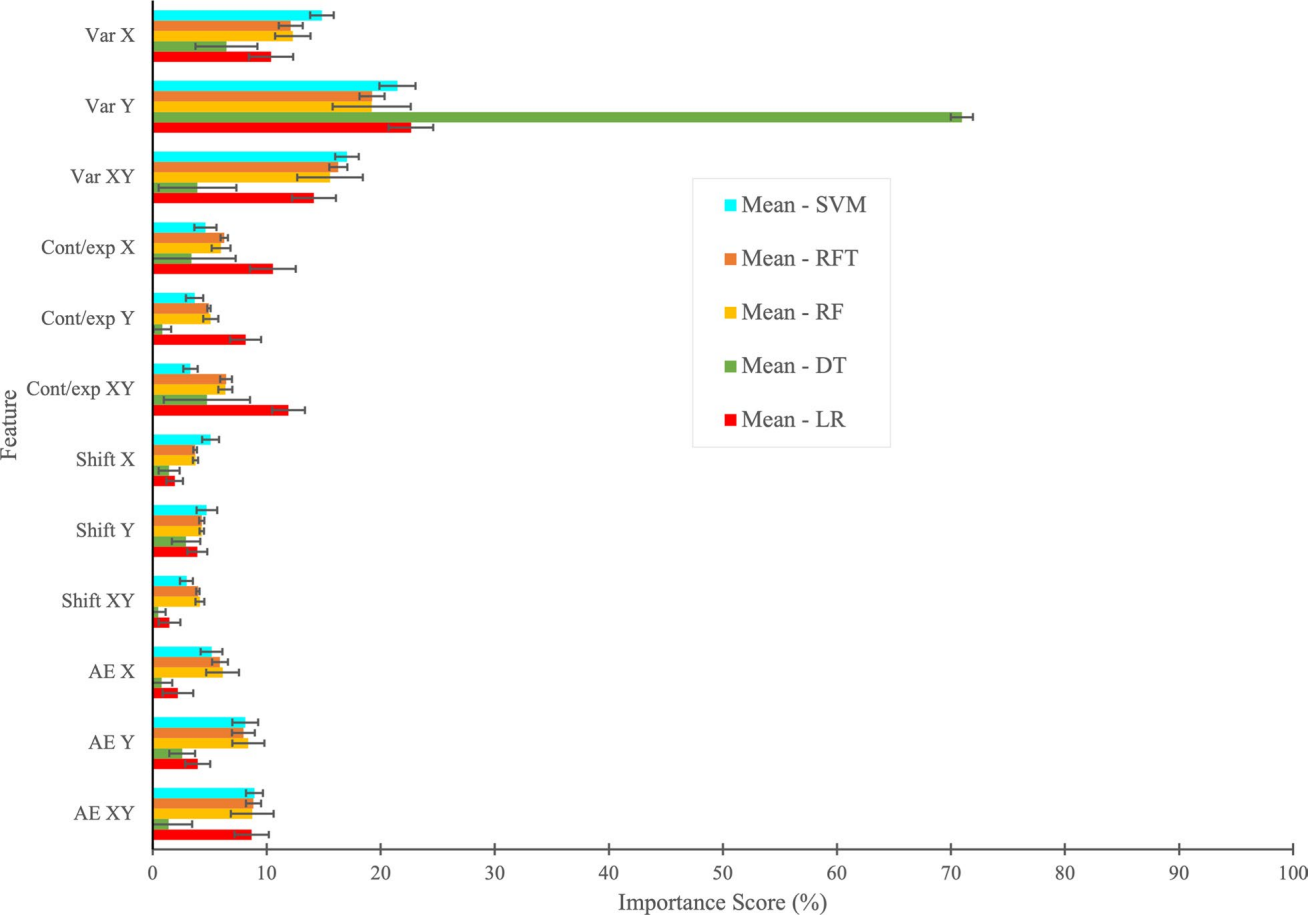
Mean and standard deviation of K-fold (K = 10) cross-validation of feature importance based on individual parameters (Var, Cont/exp, Shift, and AE) obtained from LR, DT, RF, RFT, and SVM models. Due to the complex structure of Deep Neural Network (DNN) model, we could not plot the feature importance using DNN model

*Feature Importance*: The mean and standard deviation of tenfold cross-validation of feature importance based on individual parameters (Variability, Contraction/Expansion ratio, Shift, Absolute Error) obtained using LR, DT, RF, RFT, and SVM models for the classification task is shown in Fig. 6. We were not able to plot feature importance using the DNN model because of its complex structure. We can see that different models had different feature importance scores in percentage. Across the models, some features tended to have higher feature importance scores, whereas others tended to have lower feature importance scores. For instance, we observed that *Variability Y* was the most important feature of all models for the classification task, followed by *Variability XY* and *Variability X*. The least important feature tended to be *Shift XY*, followed by *Shift X* and *Shift Y*. Although, many features contributed similarly for the classification task using LR, RF, RFT, and SVM models, the relative importance of the features appeared to be different in the DT model (see Fig. 6).

## Discussion

Proprioceptive impairment is a common consequence of stroke. Traditional clinical approaches to assess proprioception have known issues with reliability [18] and tend to rely on simplistic observer-based ordinal scales. This has led to the development of different instrumented assessment tools [31] [13, 105]. Assessments such as robotics, which can provide detailed kinematic measures, have the potential to offer new insights into the nature and severity of the proprioceptive impairments that occur after stroke. Employing machine learning techniques to explore the complex datasets that are generated by robotic assessments may prove valuable in this regard. ML techniques may help to highlight features in the data that may be important, but not obvious with other types of analyses.

The current manuscript represents our first foray into using ML techniques to classify robotic proprioceptive kinematic data from stroke survivors. We attempted to contrast the prevalence of proprioceptive deficits as identified by a previously used [31] standardized technique that relies on cut-off scores based on the 95% distribution of healthy control performance to the information derived from several different machine learning techniques. Using the cut-off score technique, we observed that the percentage of individuals classified as impaired on any given task parameter was between 10.9% and 48.4%. Variability parameters tended to detect the largest number of impaired individuals. Neuroimaging studies have shown performance on these parameters correspond with several areas of the brain including S1, posterior parietal cortex, supramarginal gyrus, superior temporal gyrus, transverse temporal gyrus, and arcuate fasciculus [106, 107]. Using an overall task score cut-off developed from the individual parameters which were equally weighted, 44% of individuals were deemed impaired. In order to more directly compare the cut-off score performance to what can be derived from ML techniques, we examined the area under the ROC curves using the overall task score and determined that accuracy of the overall task score was 85%. The machine learning techniques, on the other hand, demonstrated comparatively higher sensitivity and specificity, with areas under the ROC curves ranging from ~ 85% to 90.0% (depending on the given technique) when trying to classify whether participants did or did have a stroke based on their performance in the robot. Machine learning, by taking advantage of different parameter weightings and the relationships between parameters, performs better at classifying whether someone had a stroke. The cut-off score technique, on the other hand, tries to determine who does or does not have a proprioceptive impairment based on robotic performance (i.e., prevalence of proprioceptive deficits).

The cut-off score technique allows examination of individual kinematic variables in comparison to healthy controls (e.g., Variability_xy_), whereas the machine learning techniques, as employed, do not. In itself, individual parameters may be important for appreciating the nature of a patient’s deficits at the granular level required to precisely design an intervention. However, working with clinicians and researchers over the years, our group has been asked repeatedly to develop an overall task score that can be quickly and easily interpreted and potentially used as a primary outcome for clinical trials. The overall task score that was developed relies on summing the individual components, assuming equal weighting for each parameter, to determine a single score [108]. Despite the mathematical complexity in generating normative scores, this method is simplistic in its implementation as all parameters are equally weighted which may or may not be the most appropriate method to generate an overall score, particularly as some parameters may be highly correlated.

Machine learning techniques, as we employed them, did not calculate the prevalence of proprioceptive deficits like our cut-off score technique. Rather, we calculate and compare the accuracy, precision, recall and F1 score of the different ML techniques in attempting to classify whether someone had a stroke or not based on participant performance in the behavioural task. In general, all of the machine learning techniques we employed performed reasonably well. Perhaps this is not surprising as the machine learning techniques developed weighting values for the individual parameters (see Fig. 6), unlike the equal weighting assigned when generating overall task scores. When exposed to new data, machine learning models learn, grow, modify, change, and develop by themselves. Simply put, machine learning and deep learning techniques do this process by leveraging algorithms that learn from data in an iterative process, which is not possible using traditional data analysis methods. These differences considered, the ML models performed only slightly better than the overall task score.

Determining whether someone has had a stroke or not based on their performance in a robotic task, on the surface, may seem a bit impractical as the majority of cases of stroke already have a diagnosis based on clinical observations and confirmed with some form of neuroimaging (either computed tomography or magnetic resonance imaging). In the current manuscript, however, knowledge of the diagnosis provided a ground truth for us to test the performance of machine learning and make comparisons to the way which we have historically analyzed robotic data. While we do not see using machine learning to make the diagnosis of stroke using kinematics to be practical, our study demonstrates the potential utility of machine learning tools. Going forward, we foresee the utility of machine learning in being able to predict recovery patterns. There is also potential utility in classifying other disease conditions based on kinematics where the diagnosis is not always as clear cut as stroke (e.g., Movement Disorders) for which we have neuroimaging evidence.

In the present manuscript, the machine learning techniques we employed, for the most part, performed similarly. While these similarities were perhaps not so surprising based on other published studies that have shown relatively low variability in the results produced by different machine learning techniques [109–113], there are differences between how some of these techniques were mathematically operationalized (e.g., Logistic Regression vs. Support Vector Machines). These differences ultimately led to variability in the features/parameters the techniques considered most important (Fig. 6). In retrospect, our group would have been challenged to predict, a priori, why some features were deemed more important than others from what we have seen of previous studies based on neuroanatomic or neurophysiologic underpinnings. However, the highest variability and impairments rates in our dataset were noted to be in the parameter Variability Y which, in hindsight, explains why this feature was so important to many of the ML techniques. While overfitting can be a concern when using machine learning, we used a relatively large dataset with a cross-validation approach to minimize the risk of this. In the end, the similar performance of the different models may simply stem from the fact that the underlying dataset used was the same.

Our study is not without its limitations. The APM task we employed requires both hemispheres and as such may be more likely to be impacted by a stroke lesion, than other tasks that can be used to study proprioception such as the just noticeable difference method. Individuals with hemispatial neglect may have difficulty performing the arm position matching task [114] and the results of APM in an individual with hemispatial neglect can be difficult to disentangle from those of someone with isolated proprioceptive loss as the two impairments often co-exist. In the present study, we did not provide the ML algorithms with information about the presence or absence of hemispatial neglect as this would have ensured correct classification on the basis of the presence of the syndrome. Further, the ML techniques we employed required hundreds of datasets from individual participants which required thousands of hours to collect. In general, these techniques do not respond well to missing or unknown data, although we did not test this in the present study as we had no missing data. Like most analysis techniques used, there is always some chance of error inherent in the predictions that are made. If there is bias in the data that is used to train the machine learning techniques, this can be carried over to when a model is deployed for testing. In the present paper, we employed tenfold cross-validated datasets as this has been recommended to achieve stable results compared to traditional statistical analyses (e.g. T-test, ANOVA, Chi-Square) [115]. We then tested the model on a dataset that was not used in training to decrease the risk of bias.

As neurorehabilitation begins to incorporate more technology in both clinical practice and research settings, the opportunities for employing techniques like machine learning will continue to grow and evolve. Thoughtful application of these techniques may provide new insights into longstanding clinical problems. We could potentially see these types of techniques being used to identify prognostic factors for recovery following stroke or other disease states. There are already examples of this elsewhere in healthcare: clinical variables to predict post-stroke outcomes [113, 116–120], medical imaging diagnosis [121–124], drug discovery and manufacturing [125–127], identifying diseases and diagnosis [128, 129], outbreak prediction [130, 131], etc. Further, artificial intelligence may be helpful in guiding rehabilitation for individual patients based on their characteristics using information gleaned from thousands of patients’ journeys. Machine learning provides us with a newer set of analysis tools to enhance our understanding of data. Careful implementation could lead to significant changes in the way we carry out stroke rehabilitation in the future, but more study is needed.

## Conclusions

In this work, we applied five machine learning techniques (i.e., LR, DT, RF, RFT, and SVM) and one deep learning technique (i.e., DNN) to classify stroke patients from control participants using kinematic information from a robotic assessment of proprioception. The resulting AUC of the ROC curve can range up to 90% depending on the classifiers used. Also, we were able to find the important features, which contributed significantly to the classification task. The machine learning and deep learning models we demonstrate here can be readily applied to other clinical and medical research. We see them potentially being useful for classification using kinematics in situations where the diagnosis is, as yet, unknown and for identifying data elements (features) that are not immediately obvious as important in more classical analysis techniques. Future studies may allow the prediction of recovery using ML models.

## Supplementary Information

**Additional file 1: Supplementary Figure 1: **Histogram showing the distribution of all parameters in healthy controls and participants with stroke. The percentage on the y-axis is the participant count in each bin normalized to the number of participants with stroke (n = 429) and control (n = 465).

## Data Availability

The dataset generated for the current study are not publicly available. Data may be available from the corresponding author on reasonable request.

## Abbreviations

AE: Absolute error
APM: Arm position matching
AUC: Area under the curve
AI: Artificial intelligence
CMSA: Chedoke-McMaster stroke assessment
Cont/Exp: Contraction/expansion
CV: Cross-validation
DL: Deep learning
DNN: Deep neural network
DT: Decision tree
FIM: Functional independence measure
LR: Logistic regression
ML: Machine learning
MLR: Multiple linear regression
PPB: Purdue peg board
RASP: Rivermead assessment of somatosensory performance
ReLU: Rectified linear unit
RMSprop: Root means square propagation optimizer
RF: Random forest
RFT: Random forest with hyperparameters tuning
ROC: Receiver operating characteristics
RSS: Root-sum-square
SD: Standard deviation
SVM: Support vector machine
TLT: Thumb localization test
Var: Variability
WSPT: Wrist position sense test
