# Supplementary material for: The use of machine learning and deep learning techniques to assess proprioceptive impairments of the upper limb after stroke
Source: J Neuroeng Rehabil. 2023 Jan 27;20:15. doi: 10.1186/s12984-023-01140-9 (PMC9881388; doi:10.1186/s12984-023-01140-9)
Supplement: Supplementary file 1 — Additional file 1: Supplementary Figure 1: Histogram showing the distribution of all parameters in healthy controls and participants with stroke. The percentage on the y-axis is the participant count in each bin normalized to the number of participants with stroke (n = 429) and control (n = 465). [file 12984_2023_1140_MOESM1_ESM.docx]

Fig. 1. Histogram showing the distribution of *all parameters* in healthy controls and participants with stroke. The percentage on the y-axis is the participant count in each bin normalized to the number of participants with stroke (n=429) and control (n=465).


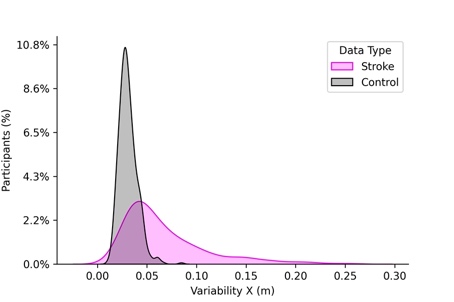

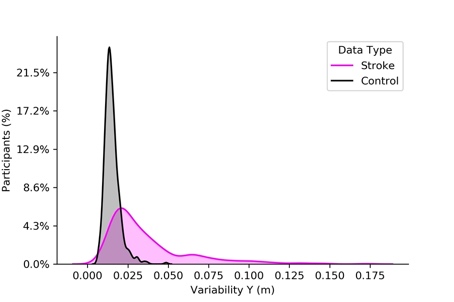

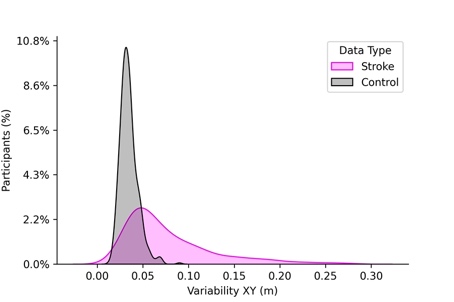


(a) Variability X (b) Variability Y (c) Variability XY


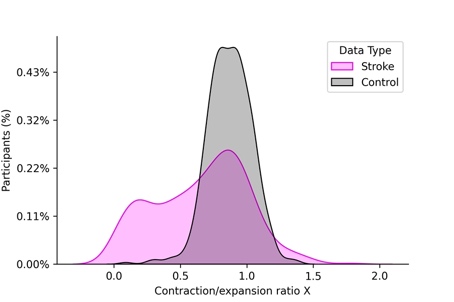

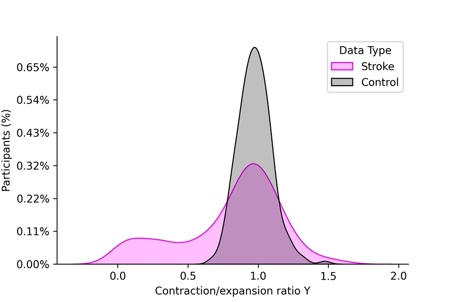

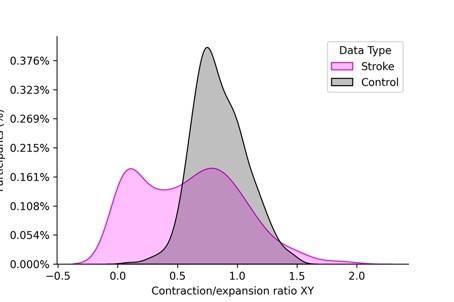


(d) Cont/Exp Ratio X (e) Cont/Exp Ratio Y (f) Cont/Exp Ratio XY


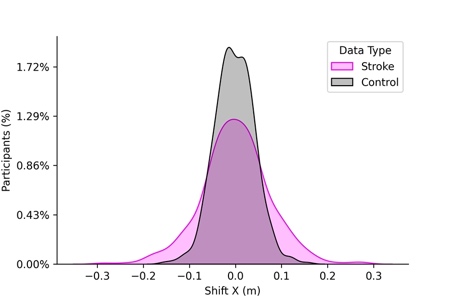

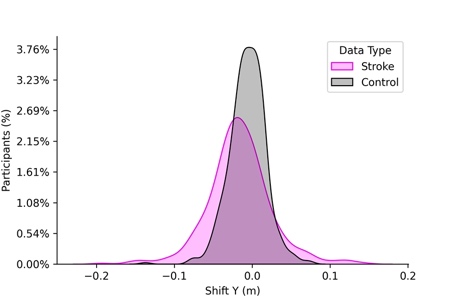

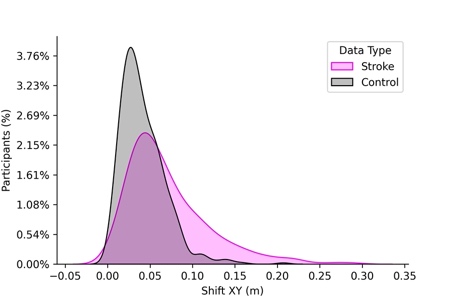


(g) Shift X (h) Shift Y (i) Shift XY


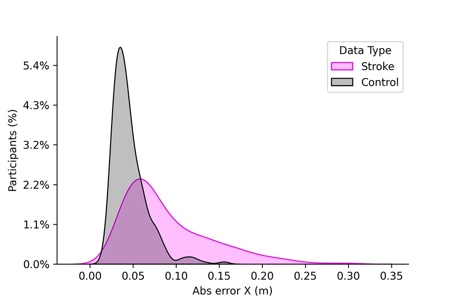

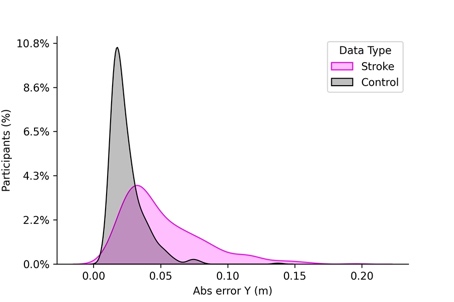

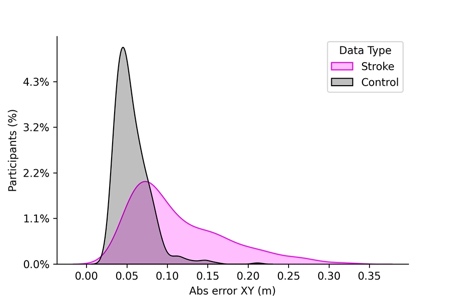


(j) Abs Error X (k) Abs Error Y (l) Abs Error XY
